# Early Application of High Cut-Off Haemodialysis for de-Novo Myeloma Nephropathy is Associated with Long-Term Dialysis-Independency and Renal Recovery

**DOI:** 10.4084/MJHID.2013.007

**Published:** 2013-01-02

**Authors:** Alhossain A. Khalafallah, Sie Wuong Loi, Sarah Love, Muhajir Mohamed, Rose Mace, Ramy Khalil, Miriam Girgs, Rajesh Raj, Mathew Mathew

**Affiliations:** 1 Launceston General Hospital, Launceston, Tasmania, Australia; 2 School of Medicine, University of Tasmania, Australia; 3 Faculty of Health and Life Sciences, Coventry University, Coventry, West Midlands, UK.

## Abstract

**Background:**

Multiple myeloma (MM) is a haematological malignancy associated with kidney injury resulting from cast nephropathy, which can be caused by monoclonal free light chains (FLC). It has been demonstrated that early reduction of FLC can lead to a higher proportion of patients recovering renal function with a better outcome, especially if high cut-off haemodialysis (HCO-HD) combined with chemotherapy is used.

**Patients and Methods:**

In this study, four cases with MM nephropathy were treated with HCO-HD and chemotherapy at a single institution during the period from August 2009 to August 2011. All of the patients presented with acute renal failure and high serum FLC. All patients underwent a bone marrow biopsy to confirm the diagnosis of MM, according to the WHO criteria. Three patients had de novo MM and one patient had relapsed light chain myeloma disease. All patients underwent HCO-HD concomitantly with specific myeloma therapy once the diagnosis or relapse of MM was established.

**Results:**

After a medial follow up of 26 months, (range, 13–36) our data showed that all patients had a significant decrease in serum FLC through HCO-HD, proving the effectiveness of HCO-HD in managing MM. De-novo MM patients restored their renal function and achieved low-level FLC early in the treatment and became dialysis-independent. One patient with relapsed myeloma remained dialysis-dependent.

**Conclusion:**

In summary, our study suggests that in myeloma nephropathy associated with light-chain MM, HCO-HD should be initiated as early as possible. At the same time a specific MM treatment should be initiated to gain control of the disease and salvage the kidneys in order to achieve dialysis-independency. Further randomized trials to confirm our results are warranted.

## Background

Multiple myeloma (MM) is a haematological malignancy most commonly associated with some degree of renal impairment and possibly acute kidney injury.^1^ The kidney injury in MM is more often caused by monoclonal free light chains (FLC) which lead to cast nephropathy.^2^–^4^ In a normal subject the immunoglobulin FLC are the by-products of immunoglobulin synthesis. The FLC are bound to the heavy chains of immunoglobulin, with only a minimal amount of unbound “free” light chains and then released in small quantities into the blood circulation. The FLC are then actively cleared by the kidneys as they are filtered through the glomerulus and reabsorbed in the proximal tubule to be broken down into amino acids which can be removed from the body safely.^5^ In myeloma patients, the clonal proliferation of plasma cells can produce FLC in quantities which exceed the normal range by hundreds to thousand fold.^5^–^8^ This overwhelms the proximal tubule’s re-absorption capacity and the excess FLC are transferred to the distal tubules. The FLC bind to a specific peptide on the Tamm-horsfall proteins where they aggregate and form casts causing cast nephropathy.^9^–^11^ Renal failure in myeloma patients can result from tubular obstruction by cast nephropathy, as well as by a severe tubulo-interstitial inflammatory process, as serum FLC can also promote proinflammatory cytokines.^12^,^13^

At diagnosis approximately 50% of patients with MM have already developed renal failure. Approximately 10% of these patients will either be dialysis-dependent or require dialysis.^9^ Patients with multiple myeloma associated with renal failure generally have a worse prognosis due to a more aggressive disease and a reduced quality of life caused by increased complications.^1^,^14^ Therefore, it is especially important in myeloma patients to find methods to reduce FLC levels in the body.

In various trials it has been demonstrated that achieving an early reduction in serum FLC levels allows renal recovery in a high proportion of MM patients and improves patient outcome.^15^,^16^ Different strategies have been tried to achieve an early reduction of serum FLC levels in MM patients. These include; plasma exchange, conventional dialysis and extended haemodialysis. Both plasma exchange and conventional dialysis may not be sufficient enough to achieve sustained removal of FLC. Studies have found that they might only remove intravascular FLC, whereas it is necessary for both intra and extra vascular FLC to be removed in order to prevent the levels of FLC re-balancing.^11^,^17^,^18^ However, extended haemodialysis using high cut-off haemodialysis (HCO-HD) in combination with chemotherapy can provide good FLC reduction. HCO membranes have large pores, with a cut off of 45kD. This provides good filtration of the kappa and lambda light chains which have molecular weights of 22kD and 45kD respectively.^19^,^20^ It is also theorized that HCO-HD removes both intra and extravascular FLC and as a result is able to remove a much greater proportion of FLC than other methods.^11^,^21^

The aim of this research was to observe the effectiveness of HCO-HD in addition to chemotherapy, in managing myeloma nephropathy in an acute setting in patients with multiple myeloma.

This study was approved by the Tasmanian Human Ethics Committee, Australia. It was registered in the Australian and New Zealand Clinical Trials Registry at http://www.ANZCTR.org.au under ACTRN 12612000024842.

## Case Series

In our study, four cases of MM nephropathy were treated with HCO-HD and chemotherapy at a single institution during the period from August 2009 to August 2011. All of the patients presented with acute renal failure and high serum FLC. All patients underwent a bone marrow biopsy to confirm the diagnosis of MM, according to the WHO criteria. Three of the cases were de-novo MM; the fourth case was relapsed MM. The patients were followed up for a median period of 26 months (range, 13–36). Male to female ratio was 1:1.

The first case (patient 1) was a 65 year old female, initially admitted with acute renal impairment and hypocalcaemia. Upon admission her creatinine was 610 μmol/L (Table 1), with a corrected calcium level of 2.99mmol/L. Her urine Bence Jones protein (BJP) showed positive and a skeletal survey showed lytic lesions at the proximal humerus and scalp. A serum electrophoresis was completed and showed elevated IgA levels of 10.3g/L and serum FLC of 7220 mg/L (Lambda), (Table 1). A bone marrow biopsy revealed a high plasma infiltration of >80%.

**Figure 1a–d:**
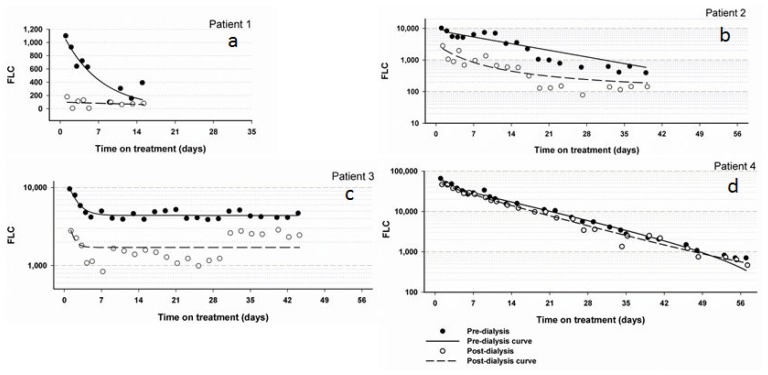
Showing FLC levels pre and post dialysis in all patients consecutively during high cut-off haemodialysis. The initial FLC with 7220 mg/L can not be shown in the graph of patient 1.

Patient 1 was commenced on thalidomide and dexamethasone, however when this did not achieve significant improvement of renal function, thalidomide was replaced by lenalidomide, and the patient was commenced on HCO-HD with baseline serum FLC of 1100 mg/L. As a result of the treatment the patient’s serum free light chain level (lambda) decreased (Figure 1.a) and her renal function significantly improved upon completion of HCO-HD. Patient 1 became thereafter dialysis-independent.

Patient 2 was a 57 year old female who presented with acute oliguric renal failure. The patient had a creatinine of 613 μmol/L and her urine BJP tested positive with lambda light chains detected.

The patient’s serum paraprotein concentration by electrophoresis was 2g/L however her serum Lambda FLC by free-lite assay was very high at 10200 mg/L (Table 1). A bone marrow aspiration revealed 90% plasma cell infiltration.

Patient 2 started treatment with bortezomib (1.3mg on days 1, 4, 8 and 11), dexamethasone (40mg on days 1–4 fortnightly). She was then commenced on HCO-HD and showed improved renal function, urine output and serum FLC (Figure 1.b). Patient 2 became dialysis-independent on discharge from hospital.

Patient 3 was a 55 year old male who was diagnosed with MM in 2005 and presented to Launceston General Hospital with acute renal failure in 2010. The patient had a creatinine level of 459 μmol/L and a serum lambda FLC level of 9620 mg/L. A repeated bone marrow biopsy confirmed progressive MM. Initially the patient’s myeloma treatment was thalidomide and dexamethasone but was changed to modified VAD (Velcade, Adriamycin and Dexamethasone) upon admission. Patient 3 was also commenced on HCO-HD but his Lambda FLC levels never dropped below 1000 mg/L (Figure 1.c). Despite several sessions of HCO-HD and a change in his chemotherapy regimen the patient remained dialysis dependant. Patient 3 was subsequently discharged from hospital with a cessation of HCO-HD and with maintenance of FLC levels with conventional dialysis in the community.

Patient 4 was a 63 year old male who presented with acute renal failure. The patient’s creatinine was 1032 μmol/L. On his second day of admission the patient was commenced on conventional haemodialysis, as his serum lambda FLC level was found to be 65500 mg/L and a bone marrow aspiration showed 40% plasma infiltration.

The patient was commenced on a treatment with thalidomide and dexamethasone in addition to the HCO-HD for up to 57 days post presentation. After two weeks, the chemotherapy was changed to modified VAD chemotherapy as the patient had a poor response to the original treatment. Patient 4 achieved dialysis-independency with a combination of chemotherapy and HCO-HD and he is now on maintenance therapy with lenalidomide.

## Discussion

The concept of HCO-HD is based on a new technology of protein permeable dialyzers which efficiently remove FLC molecules.^18^–^21^ The new haemodialysis membrane has a molecular weight cutoff similar to that of the normal kidney (approximately 65 kD).^18^ On the other hand, conventional dialysis is limited to remove only large uraemic toxins by their specific molecular weight cut-off. The recent introduction of the HCO-HD technology allows clinicians to use these membranes for the treatment of MM nephropathy with efficient elimination of high cut-off FLC molecular weight.^18^–^21^ In contrast, previously described plasma exchange (PE) removes only intravascular FLC in significant quantities; however, shortly thereafter, there is a rapid accumulation of FLC from the extra-vascular compartment into the intra-vascular compartment.^24^ Accordingly, the FLC levels return to their pre-treatment baseline without change.^24^ In contrast, HCO-HD allows an extended treatment, with subsequent redistribution of extra-vascular FLC into the intravascular compartment. By using this method, there are no significant additional side effects recorded in our cohort beyond the conventional haemodialysis. However, at least, in an ex-vivo study, there might be an increased plasma protein loss including albumin, coagulation factors, growth factors and hormones.^25^ Therefore, specific trials to examine this theory are warranted.

Our study was conducted under the assumption that; HCO-HD was an efficient way to reduce FLC concentration in MM patients; HCO-HD is more effective when the patients are diagnosed and treated early; and that chemotherapy is a necessary part of the treatment therapy.^2^,^11^

Our study showed that HCO-HD is an effective means of reducing FLC concentration in MM patients, as all of the patients, except for patient 3, showed a large decrease in their FLC concentration and improved renal function as a result of the HCO-HD. Patient 3 may not have shown the same results because he had relapsed MM and therefore HCO-HD and salvage treatment did not achieve a favorable outcome. From this a further point can be drawn that patients who achieved a low FLC level and restored their renal function early on during the treatment of MM had a better chance of becoming dialysis-independent.^2^,^11^

Patients 1, 2 and 4 had de-novo myeloma and when treated they became dialysis-independent. Patient 3 had relapsed MM and showed little response to the treatment. Patient 3 remained dialysis-dependent. This may be due to established myeloma nephropathy and possibly more renal damage, such as scarring and possible fibrosis to the kidneys and in particular the nephrons. This highlights that early treatment provides a greater likelihood of the patient becoming dialysis-independent.

This study demonstrated that the FLC concentration would rebound on successive days unless the chemotherapy was effective. Consequently whilst HCO-HD is effective in managing myeloma nephropathy, chemotherapy is also an important component of the treatment as it treats the actual disease. The chemotherapy treatment must be suited to the individual or it will not work as well. Patient 2 initially started with thalidomide and dexamethasone but showed little to no results, the chemotherapy was changed to a bortezomib containing regimen and thereafter the patient’s response was dramatically improved. This may be explained by the fact that different therapies have different effects on the myeloma kidney disease. For example bortezomib inhibits the 26S proteasome inhibitor and works down that pathway.^14^,^22^ Studies have also found that bortezomib is ideal for patients with renal injury because it reduces the serum cystatin-C level and also interferes with active nuclear factor kappa-B in renal epithelial cells and hence improves renal disease.^11^,^14^ Other drugs may not work in this way. Thus it is important to use the most appropriate course of therapy for the individual patient.

Our study also showed that HCO-HD is effective when combined with specific myeloma therapy, especially bortezomib. Without long-term effective control of MM with proper chemotherapy, patients with renal failure will inevitably have a progression of their renal nephropathy because the underlying disease has not been addressed. Thus, mechanical removal of serum FLC through HCO-HD only, is not enough without specific myeloma therapy. When HCO-HD is coupled with other therapies it can be used to manage the disease and its effects; hence improving patient’s survival. Other studies have also found that overall survival increased, from no less than 2 years to 7 years with the introduction of novel therapies.^11^ An increased efficiency of HCO-HD could further increase the overall survival while improving quality of life, as recent studies emphasise the importance of this aspect of the treatment.^23^

Multiple myeloma associated with some degree of renal impairment is responsible for approximately 1% of malignancies, 13% of these malignancies being haematological malignancies.^11^ Therefore, it is important that patients with unexplained renal failure undergo specific investigations to exclude MM, especially with the easily performed serum FLC test among other tests. If the nephropathy can be linked to MM, specific treatment for MM with HCO-HD and chemotherapy should start without delay. This would result in a favourable outcome as we demonstrated in our study.

The outcome of our study is consistent with findings from other studies.^14^–^16^ However, in our cohort of patients we found that early reduction of serum FLC led to an increased likelihood of the patient becoming dialysis-independent, with HCO-HD combined with chemotherapy being most effective. The favourable results demonstrated in cases 1, 2 and 4 should be interpreted carefully, as employing bortezomib is associated with favourable outcome in cases of myeloma nephropathy.^11^ However this was not the case in patient 3. It was most likely the combination of HCO-HD and bortezomib, which produced the favourable outcomes in patients 1, 2 and 4.

## Conclusion

Application of HCO-HD should be considered as early as possible once the diagnosis of myeloma nephropathy has been established. At the same time, a specific myeloma treatment should be initiated for each patient in order to salvage kidney function, achieve dialysis-independency and to gain good control of the disease. This may increase the chances of kidney injury being reversed and the patient becoming dialysis-independent. Accordingly, this should improve the patient’s survival and quality of life. Perhaps prospective randomised controlled trials with a larger number of patients addressing the efficacy of HCO-HD in conjunction with various treatments of MM are required.

## Figures and Tables

**Table 1:** Patient characteristics, management and follow-up of multiple myeloma patients.

	Case 1	Case 2	Case 3	Case 4
**Age at diagnosis (years) Sex**	65, Female	57, Female	55, Male	63, Male
**IgM ( on diagnosis) g/L** **Normal (0.5–2.6)**	0.2	0.1	1.4	0.2
**IgA (on diagnosis) g/L** **Normal (0.8–4.5)**	10.3	0.1	9.2	0.2
**Ig G (on diagnosis) g/L** **Normal (6–16)**	3.1	2.4	0.4	3.3
**Serum Lambda FLC (on diagnosis ) (N<19.4 mg/L)**	7220	10200	9620	65500
**Follow up Lambda FLC by end of study**	136 mg/L	404 mg/L	19500 mg/L	251 mg/L
**Serum Kappa FLC on diagnosis (N<26 mg/L)**	7	9	6.7	<6.0
**Chemotherapy lines of treatment**	Thalidomide + Dexamethasone Bortezomib	Thalidomide, Dexamethasone, Bortezomib	Thalidomide + Dexamethasone, Bortezomib Doxurubicin Lenalidomide	Thalidomide Dexamethasone Bortezomib Doxurubicin Lenalidomide
**No of HCO-HD sessions**	10	24	24	29
**Duration of the HCO-HD**	16 days	45 days	Ongoing with regular dialysis	57 days
**Creatinine (on admission)μmol/L**	610	613	459	1032
**Creatinine (on last dialysis)μmol/L**	89 (50–105)	163	507	112
**Dialysis status on follow up**	Dialysis Independent	Dialysis Independent	Dialysis Dependent	Dialysis Independent
